# Cancer Prevention in Low- and Middle-Income Countries

**DOI:** 10.1155/2017/8312064

**Published:** 2017-02-22

**Authors:** Subhojit Dey, Preet K. Dhillon, Preetha Rajaraman

**Affiliations:** ^1^Indian Institute of Public Health-Delhi, Public Health Foundation of India, Gurgaon, Haryana, India; ^2^Centre for Chronic Conditions and Injuries, Public Health Foundation of India, Gurgaon, Haryana, India; ^3^Center for Global Health, National Cancer Institute, New Delhi, India

Cancer is a rising problem across the world with a 33% increase in global cases of cancer between 2005 and 2015 [1]. The increase has been maximum in countries with the lowest development [1]. While LMICs bear a major share of the burden of cancer [1–3], very few LMICs have a comprehensive cancer prevention strategy in place. This leads to a high proportion of patients presenting at tertiary care centres at late stages of cancer, when treatment is most difficult and costly and survival is poor [4]. In the absence of adequate treatment capacity in most LMICs, patients presenting at later stages significantly increase the burden of disease. Lack of palliative care [5] compounds this situation further resulting in an unfortunate scenario where a diagnosis of cancer is equated with death in most LMICs. With reducing levels of infectious diseases and rising life expectancies, LMICs are recognizing that cancer needs to become a health priority. However, the essential steps required to prevent cancer and avoid the later consequences are still lacking emphasis. With the above scenario in mind, we had issued a call for papers that focused on cancer prevention in LMICs.

Overall, it was observed that research on cancer and especially cancer prevention in LMICs was limited [6]. Among the cancer research that is being done in LMICs, a lot of research is not of good quality. Also, most of the focus is currently on building capacity and conducting research related to cancer treatment, and cancer prevention takes a back seat, for cancer prevention requires not only facilities and human resources on the health system side but also awareness and the will and ability to pay for preventive services on the population side. Given the situation in most LMICs, both sides fall short of reaching a point where cancer prevention can be a realistic scenario. Regarding this, it is important to note the recommendations of the Breast Health Global Initiative (BHGI) which has created guidelines for breast cancer prevention while considering the economic situation of a particular nation [7]. We need to focus more on cancer prevention in LMICs, and while the job of the health providers and researchers including those who contributed to this special issue is commendable, there is immense scope for more to be done.

*Subhojit Dey* *Preet K. Dhillon* *Preetha Rajaraman*
